# A high-dose mobility booster program versus usual care for people after stroke: protocol for a pilot randomized trial

**DOI:** 10.1186/s40814-025-01613-9

**Published:** 2025-03-17

**Authors:** Katharine Scrivener, Elisha Ball, Catherine Dean, Joanne Glinsky, Louise Ada, Petra Graham, Nicholas Young, Karen Felton, Natasha A. Lannin

**Affiliations:** 1 Department of Health Sciences, Faculty of Medicine, Health and Human Sciences, Macquarie University, Sydney, Australia; 2 Faculty of Medicine and Health, The University of Sydney, Sydney, Australia; 3 Department of Mathematics and Statistics, Macquarie University, Sydney, Australia; 4 Concentric Healthcare Services, Sydney, Australia; 5 Brain Recovery and Rehabilitation Group, Department of Neurosciences, Monash University, Allied Health, Alfred Health, 99 Commercial Road, Melbourne, Victoria Australia

**Keywords:** Stroke, Mobility, High-dose, Physiotherapy, Rehabilitation

## Abstract

**Background:**

Maintaining mobility in the long term after stroke can be challenging. Furthermore, access to ongoing physiotherapy or exercise programs is limited. There is a need to investigate new models of service delivery to improve mobility in the longer term after stroke. A mobility booster program may be a solution, facilitating short-term access to physiotherapy on an as-needed basis. The aim of this project is to determine the feasibility of conducting a clinical trial of a short-term, high-dose mobility booster program (HiWalk) and measure clinical outcomes in order to estimate the power for a future efficacy trial.

**Method:**

A multi-site, assessor-blinded pilot randomized trial will be undertaken to compare HiWalk in addition to usual care with usual care alone in 50 participants. Feasibility outcomes include recruitment, adherence, and safety. Clinical outcomes include walking speed, capacity and self-efficacy at 1-month and 6-months.

**Discussion:**

A mobility booster program may be a successful way to deliver mobility training in the longer term after stroke. This pilot trial will progress the investigation of this model and assist in planning a future definitive trial. Most importantly, it will confirm the feasibility of delivering a novel high-dose, short-term booster program.

**Trial registration:**

ANZCTR (ACTRN: ACTRN12623000316606p).

## Background

Limitations to mobility are an ongoing problem after stroke. Although 70% of people after stroke regain mobility in the home [1], only 35–60% regain mobility in the community [1]. Mobility can also decline over time, with 21% of people experiencing a significant deterioration in their mobility between 1 and 3 years post stroke [1]. People after stroke often find it challenging to continue ongoing exercise, and daily physical activity is observed to be very low [2].

In many countries, including Australia, after discharge from the hospital, people after stroke are rarely offered support to improve or maintain their mobility and physical activity in the longer term [3, 4]. This means that people living with ongoing disability do not have the opportunity to achieve their preferred life goals*.* It is time to focus on how we best support people after stroke to maintain their mobility [4]. We propose a booster model, where a short-term, high-dose mobility program is offered as needed. People with stroke may have a particular mobility goal or have experienced a decline in mobility, meaning they need a boost of physiotherapy intervention, and more boosts may be required across the lifespan as further goals emerge or changes in performance occur.

### HiWalk: a mobility booster program for community-dwelling people after stroke

We have designed a novel mobility booster program called HiWalk which has two main principles—(i) high-dose training which is (ii) delivered via a self-management approach. By high-dose training, we mean motor training completed daily for 3 weeks, directed towards the achievement of a specific mobility goal. This means the participant can focus on a goal for a short time and then move on to other life activities. By self-management, we mean equipping participants with the skills and support to continue this training independently in the medium to long term. There is evidence to support the effectiveness of a self-management approach for increasing physical activity after stroke [5]. The self-management program which has been embedded into the HiWalk program is *Taking Charge after Stroke*. *Taking Charge* is a structured approach to promoting self-directed rehabilitation and self-efficacy using goal-setting as well as information provision to people after stroke [6].

Proof of concept of HiWalk has been established [7]. We found that, on average, participants trained for 185 (SD 8) min and completed 1869 (SD 543) repetitions per day while attending 79% of scheduled sessions. Acceptability of the program was excellent with all participants reporting that they would recommend the program to others. Furthermore, there were minimal adverse events and no serious adverse events. The investigator team includes a consumer representative who has evaluated the acceptability measures for the pilot trial of HiWalk.

A pilot randomised trial will allow the feasibility of conducting a definitive trial of HiWalk to be tested. The findings will aid planning for a definitive trial by understanding the recruitment, intervention and measurement procedures, as well as obtaining estimates of variability that will be used for a power calculation. This protocol for the pilot trial aims to clearly articulate the novel high-dose intervention, allowing a review of the success of intervention delivery post the pilot trial.

### Aim of the study

The aims of this study are as follows:

1. Determine the feasibility of conducting a randomised trial to investigate a mobility booster program (HiWalk) in community-dwelling people after stroke (i.e. recruiting and retaining participants, delivering the high dose intervention, the safety of the intervention and measuring clinical outcomes such as walking speed, balance, walking capacity, walking self-efficacy at 1 and 6 months), and
2. Estimate power for a definitive randomised trial based on the walking speed at 1 month.

## Method

### Design

**Fig. 1:**
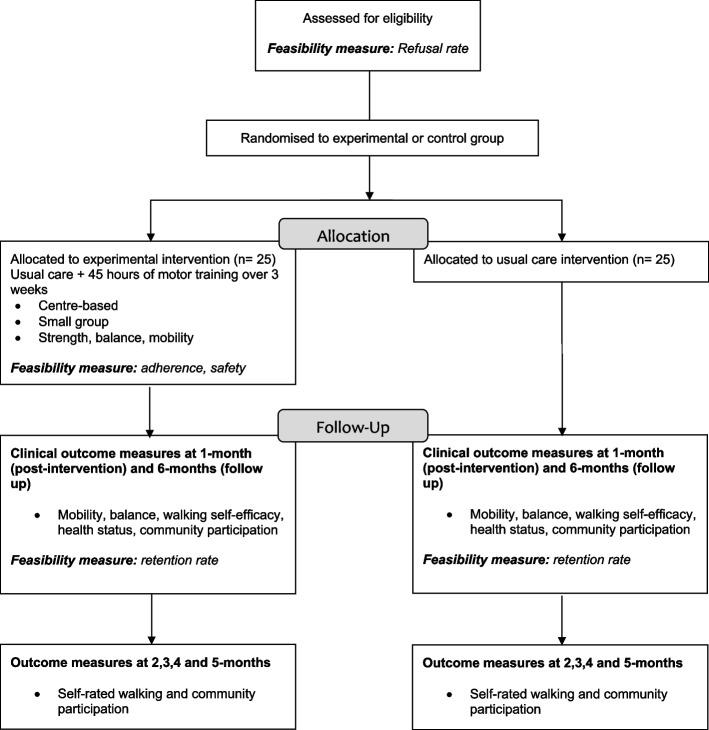
The flow of the 50 participants through the trial. Outcome timepoints and the two groups (HiWalk and usual care) are highlighted

A multi-site, assessor-blinded pilot randomized trial will be undertaken to compare HiWalk in addition to usual care with usual care alone (Fig. 1). Community-dwelling people after stroke will be recruited via advertisement, which will be distributed to relevant community groups such as stroke clubs and through professional networks. Outcome measures will be collected by a health professional who is trained in the procedures and blinded to group allocation. Participants will be asked not to discuss any aspect of the trial with the assessor to protect assessor blinding. The primary end-point will be at program completion (1 month). The secondary end-point will be at 6 months.

### Participants

Volunteers will be eligible to participate if they are as follows:

1. A community-dwelling adult with a diagnosis of stroke > 6 months and < 8 years ago
2. Able to walk without assistance from a person or aid over 10 m at a comfortable speed of 0.4–1.0 m/s
3. Willing to participate 5 days a week at a local site

They will be excluded from participating if they are as follows:

1. Unable to follow two-step instructions in English.

### Randomization

Participants will be recruited in cohorts of 16–18 participants. The cohort will be stratified according to level of walking ability with participants ranked in descending order of comfortable walking speed over 5 m and then organised into consecutive pairs. Computer-generated, independent and concealed randomization will be used to assign each participant within the pair to either the experimental or control group. Each cohort will complete intervention before the next cohort is randomized.

### Intervention

The *experimental* group will receive HiWalk for 3 weeks (up to 3 h a day, 5 days a week, totalling 45 h). HiWalk consists of motor training, i.e. strength, balance and mobility practice, delivered in a small group of 4–5 participants and 1 facilitator. Embedded throughout the program is a self-management approach where participants are guided through the *Taking Charge* program, i.e. they set individual mobility goals and are supported to complete self-directed practice [6]. HiWalk is led by a physiotherapist and sessions are facilitated by a physiotherapist, an exercise physiologist or an allied health assistant/student. A standardised assessment individualises the program which is reviewed weekly and is progressed in difficulty as appropriate. Sessions are completed in a rehabilitation gym.

Both groups may continue usual care (which may or may not include motor training) and will be recorded at baseline and 6 months. Further details of the intervention can be found in Appendix Table 3.

### Outcome measures

The outcomes include the following:

- 1. Feasibility measures (Table 1).
- 2. Clinical measures (Table 2). All clinical outcomes will be measured at baseline, 1 month and 6 months. In addition, two outcomes (self-rated walking and community participation) will be collected at 2, 3, 4 and 5 months by phone.

**Table 1:** Feasibility outcomes

*Outcome*	*Measure*	*Feasibility criteria*
Recruitment	The number of participants recruited into the study per week; proportion screened who were eligible, reasons why they were ineligible, refusal rate, e.g. number of people screened who were eligible but declined taking part in the study and the reasons why they declined. The number of participants re-measured at 1 month and 6 months, number of participants receiving and answering phone calls at 2, 3, 4 and 5 months	Refusal rate < 30% Retention rate > 85%
Intervention adherence	Average number of sessions attended per participant, reasons for non-attendance Length of each session, number of health professionals present at each session and repetitions of each activity recorded via log	Adherence > 70%
Safety	All adverse events recorded on a study adverse events form	Adverse events < 1/week
Measurement	Number of participants that all measurements were collected from	Collected from > 80%

**Table 2:** Clinical outcomes

*Outcome*	*Measure*	*Unit*
Mobility	• Walking speed: 5-m walk test to measure walking speed, step length and cadence, higher speed indicates better walking [8] • Walking capacity: 6-min walk test to measure distance covered in 6 min, higher distance indicates better walking capacity [9]	m/s Total m
Balance	Modified Step Test: number of steps in 15 s on each leg, higher score indicates better balance [10]	# steps
Walking self-efficacy	• Self-rated walking ability, higher score indicates better walking • Self-reported furthest walking distance, higher distance indicates better walking capacity • Walking self-efficacy questionnaire, higher score indicating better self-efficacy [11]	VAS 0–10 Total m 0–30
Health status	EuroQol-visual analogue scale (EQ-VAS), a higher number indicating better overall health status [12]	0**–**100
Community participation	Two purpose-designed questions about number of outings and satisfaction with level of community participation, higher number of outings indicating better community participation [11]	Satisfied (yes/no) # outings last 2 weeks

### Sample size justification

Fifty participants (25 per group) are planned to use the results of this pilot trial to inform the power analysis for a future definitive trial [13, 14]. Our target of *n* = 50 allows us to estimate anticipated retention of 85% to within a margin of error of approximately 10% with 95% confidence.

### Statistical analysis

Demographic data and feasibility outcomes will be presented using descriptive statistics. Mixed effects models will be used to adjust for the repeated measures over time. Estimated marginal means will be obtained to determine the difference in change from baseline between experimental and control groups with the multivariate-t method will be used to provide simultaneous 95% confidence intervals (CI). Trends in outcomes (self-reported walking performance and outings) will be described. Walking speed (fast) measured immediately post-intervention will be used in a power calculation for a definitive trial.

## Discussion

Programs for chronic stroke can improve the performance of mobility [15, 16]. However, most mobility programs are relatively low dose (1–3 h per week) and occur over at least 6 weeks [15, 16]. HiWalk is a mobility booster program that is high-dose (15 h per week) over a short period (3 weeks). We hypothesise that improvements in mobility are possible in this short period due to the high dose of training that will be completed. Furthermore, research to date has found that improvements after participation in a mobility program are temporary and walking ability deteriorates over time. We hypothesise that this will also occur after HiWalk and that repeat boosters will be necessary.

## Conclusion

This paper describes the protocol of a pilot randomised trial designed to investigate the feasibility of conducting a clinical trial of a high-dose mobility booster program for community-dwelling people after stroke. The data obtained in this trial will inform a future definitive clinical trial powered to detect clinically significant changes.

## Data Availability

Anonymised data from consenting participants will be stored in an open data repository managed by Macquarie University.

## Appendix

**Table 3:** Tidier checklist describing the experimental and control interventions

Checklist item	Experimental intervention	Control intervention
Brief name	HiWalk—short-term mobility booster program	Usual care
Why	Maintaining mobility in the longer term after stroke can be challenging. It is hypothesised that a short-term, high-dose, booster program may assist mobility	Pragmatic trial design
What	Participants will receive HiWalk for 3 weeks (up to 3 h a day, 5 days a week, totaling 43 h). The program consists of an individually tailored motor training program (strength, balance and task training) and specific mobility practice. Embedded throughout the program is a self-management approach	Participants may continue to participate in their usual care which may or may not include motor training
Who provided	HiWalk is led by a physiotherapist and sessions are facilitated by a physiotherapist, exercise physiologist or allied health assistant/student	
How	Booster sessions are completed face-to-face. Most sessions are completed as a small group (4–5 people). The assessment and weekly review are completed one-to-one between the participant and interventionist	
Where	Sessions are completed in a rehabilitation gym	
When and how much	Each participant receives up to 43 h of the program (up to 3 h a day, 5 days a week) over a 3-week period	
Tailoring	Each activity within the program has individualized plans developed with the therapist and the participant. Each activity has pre-planned levels of difficulty and modifications to match the activity to the participant’s ability	
Modifications	Modifications will be documented	
How well	Fidelity checks of intervention sessions will be completed	
